# Physical activity profile of the Iranian population: STEPS survey, 2016

**DOI:** 10.1186/s12889-019-7592-5

**Published:** 2019-09-13

**Authors:** Farnam Mohebi, Bahram Mohajer, Moein Yoosefi, Ali Sheidaei, Hossein Zokaei, Bahman Damerchilu, Ashkan Mehregan, Nazila Shahbal, Kamyar Rezaee, Maryam Khezrian, Ali Nematollahi Dehmoosa, Ezzatollah Momen Nia Rankohi, Mahboobeh Darman, Alireza Moghisi, Farshad Farzadfar

**Affiliations:** 1 Non-Communicable Diseases Research Center, Endocrinology and Metabolism Population Sciences Institute, Tehran University of Medical Sciences, Tehran, Iran; 2 Department of Biostatistics, Faculty of Paramedical Sciences, Shahid Beheshti University of Medical Sciences, Tehran, Iran; 3 Department of Epidemiology and Biostatistics, School of Public Health, Tehran University of Medical Sciences, Tehran, Iran; 4 Biomedical Engineering, Tonekabon branch, Islamic Azad University, Tonekabon, Iran; 5 Department of Epidemiology, Iran University of Medical Sciences, Tehran, Iran; 6 Deputy of Health, Ministry of Health and Medical Education, Tehran, Iran; 7 Endocrinology and Metabolism Research Center, Endocrinology and Metabolism Clinical Sciences Institute, Tehran University of Medical Sciences, Tehran, Iran

**Keywords:** Physical activity, Iran, Sedentary lifestyle, STEPwise approach to risk factor surveillance, Global physical activity questionnaire

## Abstract

**Background:**

Insufficient physical activity (IPA) is one of the leading causes of premature mortality through the increased burden of non-communicable diseases. From 1990 to 2017, the percentage of low physical activity attributable disability-adjusted life years (DALY) increased globally by 1.5 times and 2-fold in Iran, causing more than 1.2 million deaths worldwide and 18,000 deaths in Iran in 2017. Reports suggest that Iran, a developing middle-income country, suffers from a high level of IPA. Socioeconomic and cultural alterations along with the country’s developments expose the population to IPA risk. We aimed to describe IPA prevalence in Iran’s adult population, categorized by demographics, geographical region, and activity domains to assess the present pattern of physical inactivity and its domains in the country.

**Methods:**

In 2016, in order to represent Iran’s adult population, adult participants (n: 30541) from 30 provinces were selected using systematic proportional to size cluster sampling. Physical activity (PA) was assessed via the Global Physical Activity Questionnaire, calculating the Metabolic Equivalent of Task (MET) value in minutes per week for work, recreation, and transport domains. Insufficient physical activity (IPA) was defined according to WHO’s recommendation (less than 600 METs per week). Adjusted odds ratios of IPA associates for sociodemographic, lifestyle related variables, and metabolic risk factors were reported.

**Results:**

A high prevalence of IPA was seen in the total population (54.7%, 95%CI: 54.0–55.3) with a considerable difference between the two genders (males: 45.3% (95%CI: 44.3–46.3); females: 61.9% (95%CI: 61.0–62.7)). Work-related activity was the domain with the greatest percentage of total PA, whereas, both genders lacked recreational activities. In our findings, being female, a housekeeper, younger and living in urban areas were significantly associated with higher levels of IPA. Moreover, insufficient fruit and vegetable consumption, lack of alcohol consumption, having a personal vehicle, and finally, having a medical history of diabetes were significantly associated with the presence of IPA in our population. Among the study population, 33.6% (95%CI: 33.0–34.2) had at least 4 h of sedentary behavior in a typical day.

**Conclusions:**

Widespread IPA among the Iranian adult population is of major concern. In our findings, we observed a considerable gap in the prevalence and pattern of IPA between the two genders. Additionally, IPA was associated with living in urban areas, unhealthy lifestyle habits and a history of other metabolic risk factors. Thus, a prompt initiative for population-specific actions should be taken.

**Supplementary information:**

**Supplementary information** accompanies this paper at 10.1186/s12889-019-7592-5.

## Introduction

Insufficient physical activity (IPA) increases the risk of many chronic diseases including diabetes, cardiovascular diseases (CVD) and cancer [1–6]. IPA is directly responsible for a decrease in life expectancy [7]. The Global Burden of Disease Study (GBD) indicated that from 1990 to 2017, the percentage of low physical activity attributable disability-adjusted life years (DALY) increased globally by 1.5 times and 2-fold in Iran [8]. Moreover, in 2017, low physical activity was responsible for more than 1.2 million deaths worldwide and 18,000 deaths in Iran [8]. Conversely, a higher level of physical activity has benefits in different aspects of health, including the prevention of many non-communicable diseases (NCDs) [9] as well as improved functional and cognitive performance in daily life [10]. The World Health Organization (WHO) has proposed the minimum recommended physical activity levels and insufficient physical activity (IPA) below these thresholds is one of the leading risk factors responsible for death worldwide [11].

Iran has been among the countries with medium to high IPA in global reports. In 2011, national reports revealed an IPA rate of 39.1% among the population [12]. An increasing pattern of sedentary lifestyle and IPA in a variety of settings have been previously reported [13, 14], and considering IPA catastrophic economic burden on the health system [15], and the documented potential for effective interventions, there is a pivotal need for a comprehensive and up-to-date report in this regard to determine future health policies. Furthermore, timely reaction requires an updated and detailed report of the current status, in order to provide an opportunity to assess and evaluate the impact of previous policies, as no official report is available.

The present study aimed to describe the prevalence of IPA among Iran’s adult population, categorized by demographics, geographical region, and activity domains to assess the present pattern of physical inactivity and its domains in the country. Furthermore, enlightening the present patterns could prepare fertile ground for precise and effective national policy planning, leading to increased physical activity and as a result, decreased prevalence of attributed NCD risk factors.

## Methods

Initiated in 2000, the WHO STEPwise approach to risk factor Surveillance (STEPS), developed a set of feasible and standardized methods for data collection, processing and reporting on a global scale [16]. The STEPS study is an ongoing sequential large-scale cross-sectional population-based surveillance of NCDs risk factors. Previous studies based on WHO STEPS have been surveyed in Iran in 2005, 2006, 2007, 2008, 2009, and 2011 [17]. This study was part of the most recent STEPS project, carried out in 2016 [18]. The questionnaire was first established in 2005. The Persian translation of earlier studies’ STEPS questionnaires was thereafter revised to evaluate the questions’ consistency and assess their validity and reliability. Consequently, an improved version was developed to investigate the demographic and behavioral data, history of metabolic risk factors and injuries, healthcare utilization, and screening programs and treatment. Step 1 of the study was to interview the participants and have them fill the questionnaire. Step 2 included physical assessment (anthropometric measures, blood pressure, and pulse rate). Step 3 consisted of lab assessments (total cholesterol, glucose, HDL-C, Alanine Aminotransferase (ALT), triglycerides, Hemoglobin A1c, urine sodium, and urine creatinine) exclusively performed on a subgroup of adults over the age of 25. Further details of the protocol were comprehensively explained elsewhere [18].

### Sampling

In order to conduct proportional to size sampling, a systematic random sampling frame was designed. From both rural and urban areas of all 30 provinces of Iran, 31,050 participants in 3105 clusters were selected (each including 10 participants). The variables considered in the representative samples were age, gender, place of residence (rural/urban) and province. To estimate the minimum sample size with a 95% confidence level, calculations were based on the province of Ilam, as it was the most sparsely populated with 384 samples. The sample size in other provinces was estimated based on the population ratio of each province to Ilam as reference. After estimation, 10% was added to the sample size of each province to control non–response errors and consider the effect of sampling design. To reduce study costs, only half of the calculated sample size was considered in provinces with more than 800 clusters, while the weight applied in the subsequent statistical analysis was doubled to compensate for the halved sample size.

### Study population

Of the 31,050 participants chosen from urban and rural areas of Iran’s 30 provinces via systematic proportional to size cluster sampling, representative of the Iranian adult population, a total of 30,541 individuals participated in the study (non-response rate = 1.64%). The main reason of the non-response was the subjects’ unwillingness to participate in the research or unavailability of subject at defined postal address at the randomized sampling after three consecutive tries. At the time of data collection, all study samples were over 18 and residents of Iran. Individuals who were willing to participate and completed the written informed consent forms were interviewed and further assessed. A total of 26,541 subjects were between 18 and 64 years of age. The remaining participants were elderly people over the age of 65.

### Physical activity assessment

The Global Physical Activity Questionnaire (GPAQ) has been used in WHO STEPS methods to assess physical activity and sedentary behavior [19]. We utilized GPAQ version 2.0, which comprehensively assesses physical activity of the population. It categorizes physical activity into work, transport and recreation (the domains of physical activity), via face to face interviews with all eligible participants based on the STEPS protocol. The reliability and validity of GPAQ has been assessed in many countries with reliability coefficients demonstrating moderate to substantial strength [20, 21]. Data collection processes were validated in the STEPS 2016 conducted in Iran [18].

Metabolic equivalent of task (MET) scores were calculated and used as the measurement unit for domains of physical activity, based on the GPAQ Instrument and Analysis Guide v2. One MET is defined as 1 kcal/kg/hour, equivalent to the energy cost of sitting quietly while consuming 3.5 ml/kg/min of oxygen. Four METs and 8 METs were assigned to the time spent doing activities of moderate- and vigorous-intensity, respectively. Sufficient physical activity was defined as any combination of physical activities that exceeds 600 METs per week, according to the February 2017 updated version of the WHO physical activity fact sheet. This included at least 150 min of moderate-intensity physical activity throughout the week, or at least 75 min of vigorous-intensity physical activity throughout the week, or an equivalent combination of both moderate- and vigorous-intensity activities [22]. Our primary outcome of interest was the point prevalence of IPA. Sedentary lifestyle was defined as spending more than 4 h sitting or reclining on a typical day. Other measures of physical activity used in this article, are described in detail in Table 1.

**Table 1:** Assessment and levels variables regarding physical activity, its domains, demographic, and risk-related factors used in the analysis

Physical activity		
Variable name	Variable assessment	Variable levels/unit
Total physical activity	The sum of the total MET minutes of work-related, transport-related, and recreational activity	MET-minutes per week
Vigorous intensity physical activity	Self-reported physical activity that significantly increases the heart beat rate and continues for at least 10 min, in either domains of work or leisure	Minutes
Total work-related physical activity	Total work-related physical activity in minutes per week	Minutes per week
Total transport related physical activity	Total transport related physical activity in minutes per week	Minutes per week
Total recreational physical activity	Total recreational related physical activity in minutes per week	Minutes per week
Work no activity	Self-reporting of no moderate or vigorous work-related physical activity	Yes, No
Transport no activity	Self-reporting of no moderate or vigorous transport-related physical activity	Yes, No
Recreational no activity	Self-reporting of no moderate or vigorous recreational physical activity	Yes, No
Sedentary behavior	Self-report of total time spent in sedentary more than 4 h per day	Yes, No
Sociodemographic		
Sex	Report of the interviewer proofed by ID card	Male, Female
Age	Report of the interviewee proofed by ID card and categorized by WHO recommendations.	18–24, 25–34, 35–44, 45–54, 55–64, 65–69, 70 ≤
Residence area	Established geographical divisions of the country	Rural, Urban
Marital status	Self-report	Married, Never married, Divorced or Living alone, Widow
Occupation	Self-report	Non-paid or self-paid, Employee of governmental or non-governmental organization, Worker in a public or private section, Student, Soldier, Unemployed or Retired, Housekeeper
Education	Self-report of number of completed education years	Illiterate, 1–6 years, 7–12 years, > 12 years
Wealth index	Reduction of dimension in factor of wealth with PCA (Principle component analysis) model	1st (poorest), 2nd, 3rd, 4th, and 5th (richest) quintiles
Life style		
Appropriate fruit and vegetable consumption	Self-report of less than 2 units of fruit and 3 units vegetable consumption per day	Yes, No
High salt intake	Self-report of equal or more than 5 g salt consumption per day	Yes, No
Smoking	Self-report of ever daily smoking	Ever smoker, never smoker
Alcohol consumption	Self-report of ever alcohol consumption in the last month	Yes, No
Injury	Self-report: any accident resulting in outpatient emergency room management or hospital admission in the last 12 months	Yes, No
Personal car ownership	Self-report of ownership of a personal car	Yes, No
Metabolic risk factors		
Hypertension	Self-report of past or present history of hypertension	Yes, No
Past medical history of cardiovascular disease	Self-report past or present history of the myocardial infarction or cerebrovascular event in the past 12 months	Yes, No
Diabetes mellitus	Fasting plasma glucose more than 126	Yes, No
Dyslipidemia	Serum total cholesterol levels ≥200 mg/dl (≥5·2 mmol/liter)	Yes, No
Abdominal obesity	Waist circumference more than 102 cm in men and 88 cm in women	Yes, No
BMI (body mass index)	Calculated as measured weight in kilograms divided by square of measured height in meters	> 18·5: Underweight, 18·5–25: Normal, 25–30: Overweight, 30≤: Obese

### Data analysis

All analyses were performed with STATA version 14. The *t*-test and ANOVA were used for comparing continuous variables. To evaluate associations with categorical variables, a logistic regression model was applied. Data cleaning was done according to WHO STEPS surveillance manual [23] and GPAQ Instrument and Analysis Guide v2 [19].

Total physical activity (TPA) and descriptive results were presented twice; once for the whole population, who were 18 to over 70 years of age [18], and once more for the 18 to 64-year-old subjects. The percentage of each domain, including work, transport and recreation was also presented. To present the pattern of IPA and its differences among the samples, the IPA prevalence was reported in different subgroups of the study population based on the covariates presented in Table 1. Associations and further analyses were only done for adults between the ages of 18 and 64. The associates consisted of the covariates presented in Table 1, with the exception of PA. In the inferential analysis, the odds of IPA in different subgroups of explanatory variables were investigated and compared to the base group. The IPA odds ratio was adjusted separately and concurrently for sociodemographic variables, metabolic risk factors, and lifestyle related characteristics as shown in Table 1. The results were stratified by gender in all reports. In all statistical analyses, weighting was performed considering: 1) the weighted sampling of provinces with ≥800 clusters; 2) The missing and non-response data within each cluster of data; 3) weighting based on province population, considering the missing data of one province (Qom) for the sake of representativeness; 4) weighting based on the age and gender of each population distribution for the sake of representativeness; 5) separate weighting for the missing/non-response question, within answers/measurements of each subject; 6) the missing, non-response, or data not acquired within each category of the questionnaire, body measurements, and laboratory tests to consider the difference in subjects who had data for each of these categories. When evaluating or inserting diabetes in the model, 18 to 24-year-old subjects were excluded because based on the study protocol, their blood was not sampled.

### Role of the funding source

The funding source had no role in the study design, data collection, analysis, data interpretation, manuscript writing, and the decision on paper submission.

## Results

### Subjects

A total of 26,541 adults between the ages of 18 and 64 (males: 47.3%; females: 52.6%; mean age: 40.01) were included in the study. Most of the population were educated (9.4% illiterate), urban residents (71.5%), female housekeepers (44.8%) and self-employed males (25.0%).

### Insufficient physical activity scale of Iran’s population

**Fig. 1:**
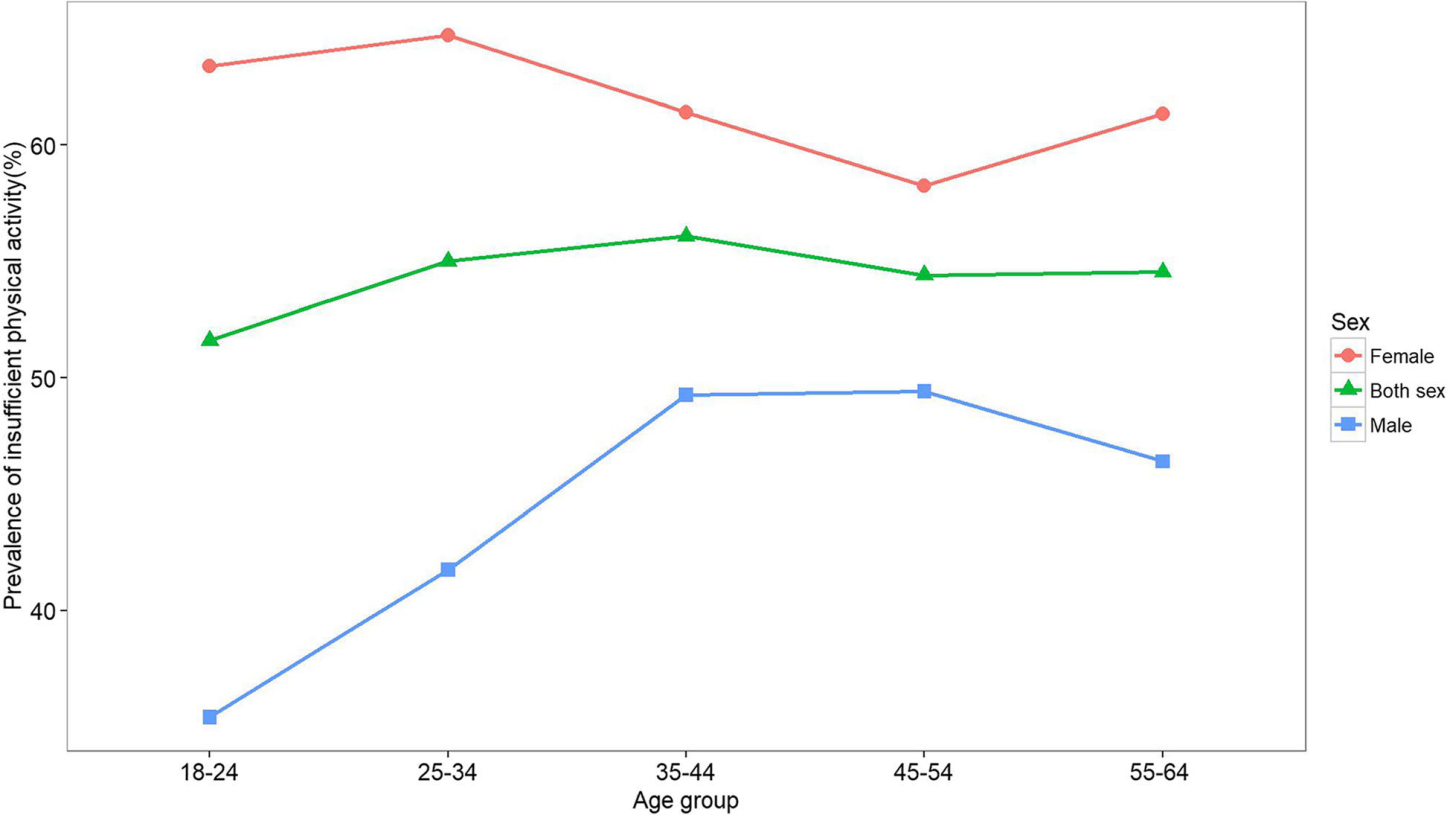
Prevalence of insufficient physical activity among the Iranian adult population by gender and age groups

The mean IPA prevalences for the whole population, males, and females were 54.7% (95%CI: 54.0–55.3), 45.3% (95%CI: 44.3–46.3) and 61.9% (95%CI: 61.0–62.7), respectively. The odds of IPA in females was nearly two times greater than that of males (OR 1.95, 95%CI: 0.48–0.53). As expected, IPA had the lowest prevalence in the 18–24-year-old age group (51.6, 95%CI: 49.6–53.6) and the highest prevalence in the 35–44-year-old age group (56.1, 95%CI: 54.7–57.4). The age pattern of IPA prevalence was influenced by gender; while IPA increased in the youngest to the middle-aged male population, it decreased in females. This resulted in a converging pattern between males and females as their age increased. In all age groups, IPA prevalence was higher in females. The highest difference was apparent in the youngest adults whereas the lowest difference was seen in 45 to 54-year-old subjects (Fig. 1 and Table 2).

**Table 2:** Assessment and levels of physical activity measures in Iranian adult and elderly population

Age categories	Total Frequency (95% CI)	Male Frequency (95% CI)	Female Frequency (95% CI)
Population with insufficient physical activity			
18–24	51·6% (49·6–53·6)	35·4% (32·4–38·5)	63·4% (60·8–65·9)
25–34	55·0% (53·7–56·3)	41·8% (39·8–43·7)	64·7% (63·1–66·3)
35–44	56·1% (54·7–57·4)	49·3% (47·2–51·3)	61·4% (59·7–63·1)
45–54	54·4% (53·0–55·8)	49·4% (47·2–51·6)	58·2% (56·4–60·1)
55–64	54·5% (52·9–56·1)	46·4% (44·0–48·8)	61·3% (59·2–63·4)
65–74	62·4% (60·4–64·5)	49·5% (46·4–52·7)	72·7% (70·2–75·2)
75+	73·8% (71·5–76·1)	62·6%(59·2–66)	87·2% (84·7–89·8)
18–64	54·7% (54·0–55·3)	45·3% (44·3–46·3)	61·9% (61·0–62·7)
18–75+	56·3% (55·7–57·0)	46·8% (45·9–47·7)	63·9% (63·1–64·7)
Population with no activity at work			
18–24	75·1% (73·3–76·9)	68·4% (65·4–71·4)	79·9% (77·8–82·1)
25–34	71·8% (70·7–72·9)	61·1% (59·2–63·1)	79·6% (78·3–81·0)
35–44	69·6% (68·4–70·9)	61·2% (59·2–63·2)	76·2% (74·7–77·7)
45–54	71·8% (70·5–73·0)	65·1% (63·0–67·2)	76·9% (75·3–78·5)
55–64	75·0% (73·6–76·4)	69·6% (67·4–71·7)	79·5% (77·8–81·2)
65–74	80·1% (78·5–81·8)	75·1% (72·4–77·7)	84·2% (82·2–86·3)
75+	87·7%(86·0–89·4)	82·9% (80·2–85·5)	93·6% (91·7–95·5)
18–64	72·0% (71·0–72·0)	64·0% (63·0–65·0)	78·0% (77·0–78·0)
18–75+	73·7% (73·1–74·2)	66·4% (65·5–67·3)	79·4% (78·8–80·1)
Population with no activity at transport			
18–24	40·9% (38·9–42·9)	36·6% (33·5–39·7)	44·0% (41·4–46·6)
25–34	44·2% (43·0–45·5)	43% (41·0–44·9)	45·1% (43·5–46·8)
35–44	44·8% (43·5–46·2)	46% (44·0–48·1)	43·9% (42·1–45·7)
45–54	43·5% (42·1–44·9)	44·5% (42·3–46·6)	42·8% (40·9–44·6)
55–64	40% (38·5–41·6)	36·1% (33·9–38·4)	43·3% (41·1–45·4)
65–74	47·9% (45·8–50·0)	56·6% (53·8–59·4)	37·1% (34·0–40·1)
75+	62·5% (60·0–65·0)	77·6% (74·3–80·8)	50·0% (46·5–53·5)
18–64	43·0% (42·0–43·0)	42·0% (41·0–43·0)	43·0% (43·0–44·0)
18–75+	44·6% (43·9–45·2)	42·2% (41·3–43·1)	46·5% (45·6–47·3)
Population with no recreational activity			
18–24	69·9% (68·0–71·8)	51·5% (48·3–54·7)	83·2% (81·2–85·2)
25–34	77·4% (76·3–78·4)	65·1% (63·3–67·0)	86·3% (85·2–87·5)
35–44	82·4% (81·4–83·4)	75·5% (73·7–77·2)	87·9% (86·7–89·0)
45–54	85·8% (84·8–86·8)	80·6% (78·9–82·3)	89·8% (88·6–90·9)
55–64	88·7% (87·7–89·7)	84·8% (83·1–86·5)	92·0% (90·8–93·2)
65–74	91·4% (90·2–92·6)	88·1% (86·0–90·2)	94·0% (92·7–95·4)
75+	95·7% (94·6–96·7)	93·5% (91·7–95·3)	98·3% (97·2–99·3)
18–64	81·0% (80·0–81·0)	72·0% (71·0–73·0)	87·0% (87·0–88·0)
18–75+	83·0% (82·6–83·5)	75·5% (74·7–76·3)	88·9% (88·4–89·5)
Population with sedentary behavior			
18–24	38·8% (36·8–40·8)	33·5% (30·5–36·6)	42·6% (40·0–45·2)
25–34	33·1% (31·9–34·3)	30·2% (28·4–32·0)	35·2% (33·6–36·8)
35–44	30·5% (29·2–31·7)	29·2% (27·3–31·1)	31·5% (29·9–33·2)
45–54	32·9% (31·5–34·2)	32·6% (30·6–34·6)	33·1% (31·3–34·9)
55–64	36·7% (35·1–38·2)	37·0% (34·7–39·3)	36·4% (34·3–38·5)
65–74	43·5% (41·4–45·6)	40·5% (37·4–43·6)	46·0% (43·2–48·8)
75+	52·6% (50·0–55·1)	49·1% (45·6–52·6)	56·7% (52·9–60·5)
18–64	33·0% (33·0–34·0)	32·0% (31·0–32·0)	34·0% (34·0–35·0)
18–75+	35·5% (34·9–36·1)	33·9% (33·0–34·8)	36·8% (36·0–37·6)
Total Calculated METs	Mean ± SE	Mean ± SE	Mean ± SE
18–24	1847·0 ± 64·8	2854·2 ± 123·5	1114·1 ± 58·8
25–34	1767·0 ± 39·9	2703·4 ± 77·8	1079·8 ± 34·3
35–44	1785·0 ± 42·6	2482·5 ± 79·9	1243·5 ± 41·0
45–54	1772·7 ± 45·2	2357·0 ± 84·7	1321·9 ± 44·5
55–64	1619·7 ± 45·0	2173·1 ± 79·9	1156·9 ± 46·0
65–74	1292·1 ± 54·1	1940·9 ± 102·4	772·3 ± 47·6
75+	864·0 ± 52·2	1288·8 ± 85·3	352·3 ± 44·3
18–64	1756·5 ± 20·4	2500·1 ± 38·5	1885·6 ± 19·2
18–75+	1669·2 ± 18·5	2371·5 ± 34·4	1114·6 ± 17·4
Percentage contribution of vigorous PA in total MET	Mean % ± SE	Mean % ± SE	Mean % ± SE
18–24	20.75 ± 0.91	33.55 ± 1.47	8.48 ± 0.87
25–34	18.1 ± 0.54	29.13 ± 0.92	7.89 ± 0.50
35–44	15.98 ± 0.55	25.4 ± 0.95	7.70 ± 0.54
45–54	13.58 ± 0.54	21.14 ± 0.94	7.08 ± 0.55
55–64	10.21 ± 0.51	15.01 ± 0.87	5.43 ± 0.54
65–74	7.64 ± 0.65	10.67 ± 1.01	4.06 ± 0.72
75+	5.57 ± 0.84	5.87 ± 0.95	4.76 ± 1.71
18–64	15.64 ± 0.26	24.68 ± 0.45	7.33 ± 0.26
18–75+	14.69 ± 0.24	25.56 ± 0.41	7.06 ± 0.24

*CI* Confidence Interval, *SE* Standard deviation, *PA* Physical activity, *MET* Metabolic equivalent of task

### Domains of physical activity

In all age groups, the largest domain of physical activity was work (53.7%), followed by transport (33.6%) and recreation (12.8%). The pattern was almost the same for both genders. However, the work-domain’s highest contribution was seen in 35–44-year-old individuals, overall, but its percentage was higher in men (64.7%) as compared to women (53.1%). Additionally, the work-domain MET value was highest for both genders in this age group. By contrast, the lowest transport-domain MET value was that of males (Additional file 1: Figures S1 and S2). The mean work-domain MET value for males was more than two times greater than that of females. Overall, female and male individuals between 55 and 64 years of age had the highest percentage of TPA in the transport-domain. Females between the ages of 55 and 64 years were the only group in which the transport-domain TPA was higher than the work-domain TPA. Overall, the highest percentage of recreation-domain TPA was seen in both male and female 18–24-year-olds. The youngest males were the only group in which the percentage of the recreation-domain TPA was higher than the transport-domain TPA. Moreover, this age group had the lowest IPA prevalence in males and overall, but considerably high IPA prevalence in females (Fig. 1). In all the domains, zero physical activity was more common in females. However, the highest difference was observed in recreational physical inactivity (44% in males vs. 80% in females).

### Intensity of physical activity

From the total METs of Iran’s adult population, 15.64% ± 0.26 was attributed to vigorous-intensity PA. As expected, the percentage of vigorous PA decreased with an increase in age from 20.75% in 18–24-year-old subjects to 10.21% in 55–64-year-old individuals. However, the percentage of vigorous PA was nearly three times greater in male subjects, irrespective of their age group.

### Sedentary lifestyle

**Fig. 2:**
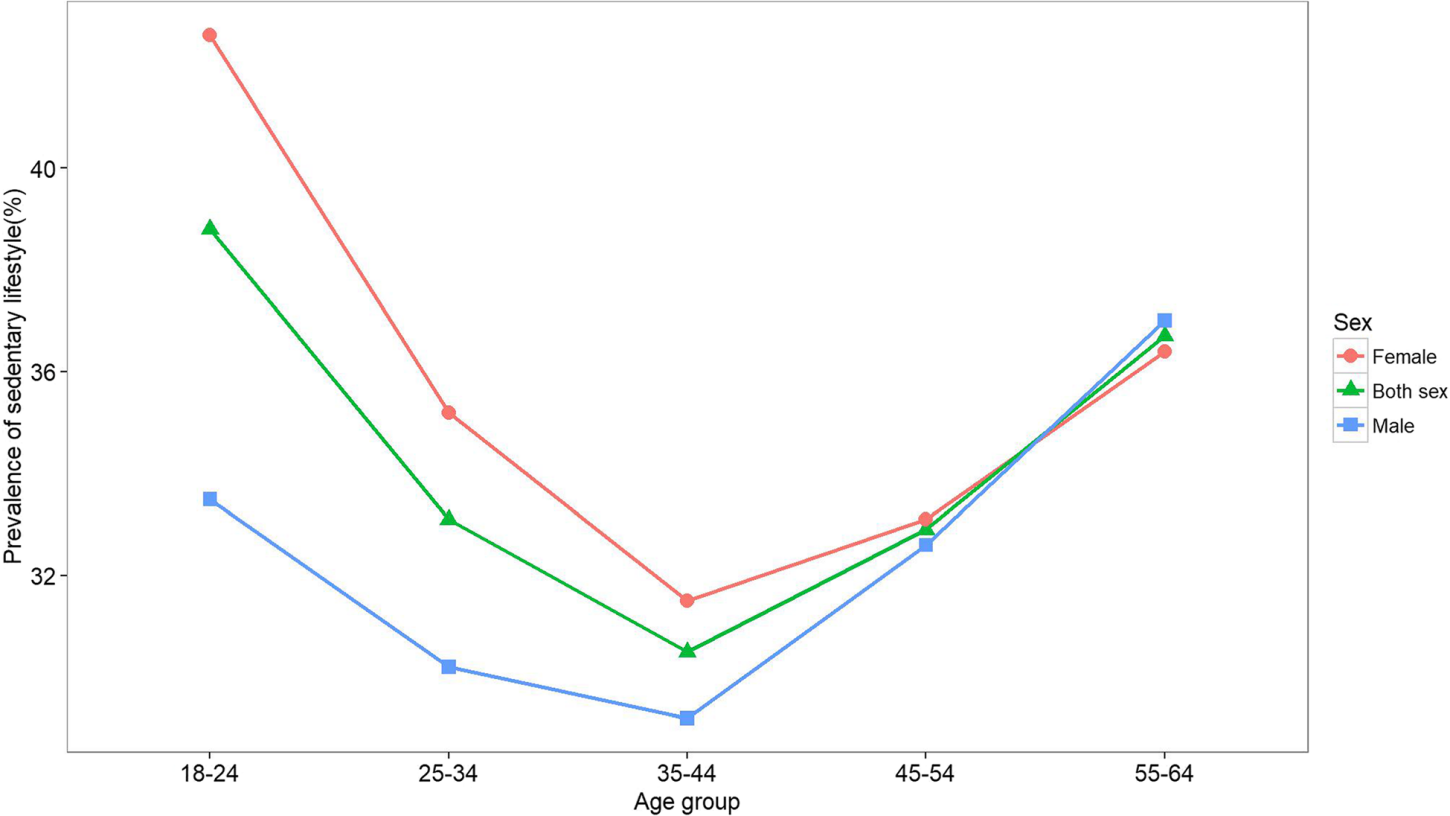
Prevalence of sedentary lifestyle in the Iranian adult population by gender and age groups, defined as more than 4 h of sedentary behavior in a typical day

Overall, 33.6% (95%CI: 33.0–34.2) of the population had at least 4 h of sedentary behavior (males: 32.0% (95%CI: 31.0–32.9); females: 34.9% (95%CI: 34.0–35.7)) in a typical day. The highest percentage of sedentary lifestyle was among 18–24-year-old individuals (38.8% (95%CI: 36.8–40.8)) and the same in females (42.6% (95%CI: 40.0–45.2)). However, the highest percentage of sedentary lifestyle (37.0% (95%CI: 34.7–39.3)) was observed among the oldest males. In all age categories, the prevalence of a sedentary lifestyle was higher in females, except for the 45–54 and 55–64 age groups. Details are presented in Table 2 and Fig. 2.

### Associates of insufficient physical activity

**Fig. 3:**
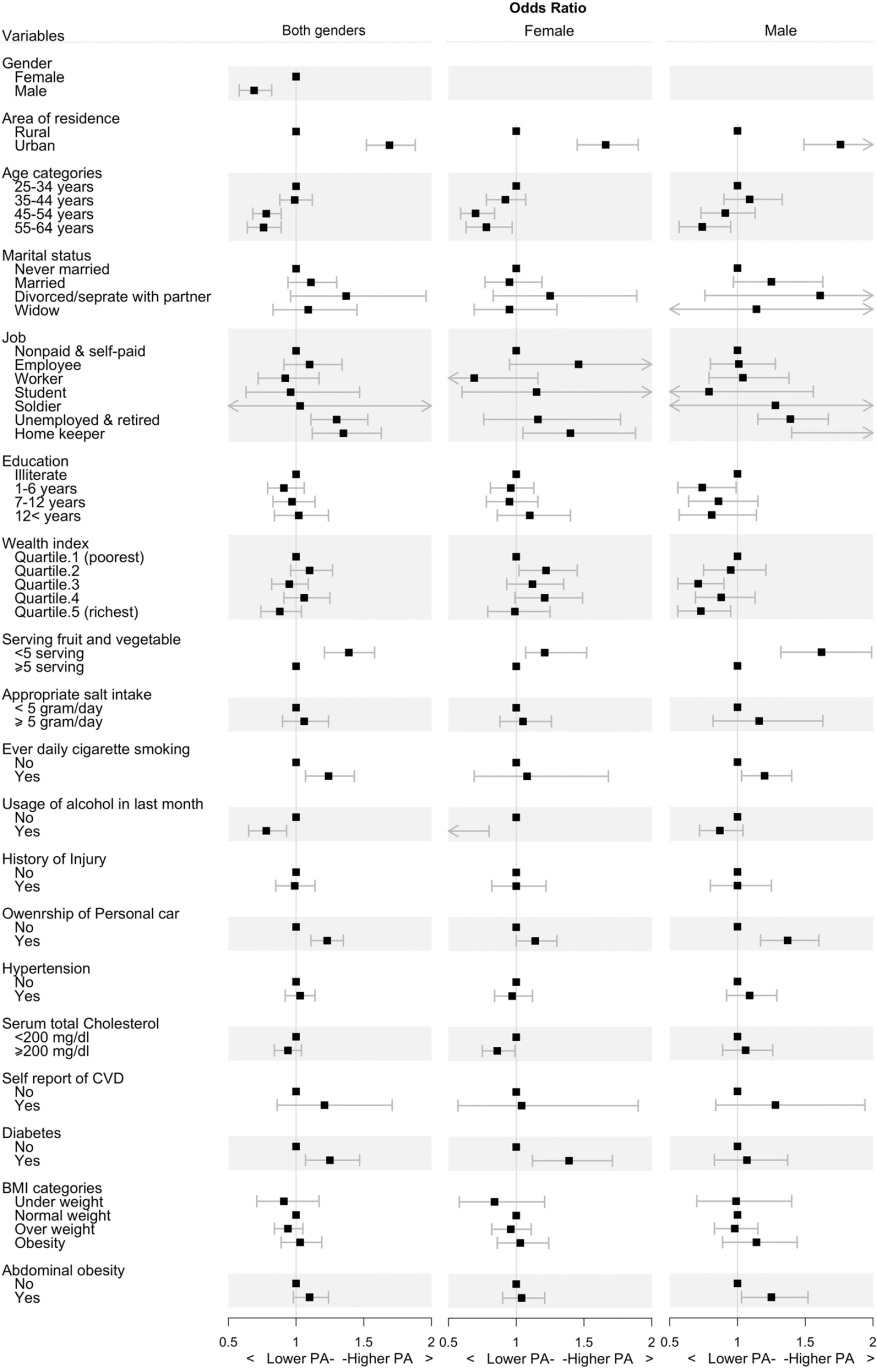
Forest plot of insufficient physical activity correlates in the entire Iranian adult population and gender subgroups

Table 3 demonstrates the crude and adjusted odds ratios (ORs) of covariates categorized by gender. The gender-specific prevalence of each variable can be found in Additional file 1: Table S1. In general, the adjusted odds of IPA were lowest among: males (vs females), oldest (vs youngest: 0.76 (95%CI: 0.64–0.89), P: 0.001), never-marrieds (vs marrieds: 1.11 (95%CI: 0.94–1.3), P: 0.219), rural residents (vs urban residents: 1.69 (95%CI: 1.52–1.88), P: < 0.001), workers (vs non-paid and self-paid: 0.92 (95%CI: 0.72–1.17), P: 0.503), individuals with 1–6 years of completed study (vs illiterate: 0.91 (95%CI: 0.79–1.06), P: 0.226), and richest (vs poorest 0·88: (95%CI: 0.74–1.04), P: 0.137), as presented in Fig. 3. Lower adjusted odds of IPA were observed in individuals who had: sufficient fruit and vegetable consumption per day (insufficient vs sufficient consumption: 1.4 (95%CI: 1.23–1.59), P: < 0.001), consumed less than 5 g of salt per day (more vs less than 5 g: 1.06 (95%CI:: 0.9–1.24), P: 0.5), reported to have never smoked (some vs never: 1.24 (95%CI: 1.07–1.43), P: 0.004), consumed alcohol in the last month (some vs never: 0.78 (95%CI: 0.65–0.93), P: 0.005), a history of injury (positive vs negative: 0.99 (95%CI: 0.85–1.14), P: 0.861), and who did not have a personal vehicle (positive vs negative: 1.23 (95%CI: 1.11–1.35), P: < 0.001). In general, the following were associated with lower adjusted-odds of IPA: normal BMI (underweight vs normal BMI: 0.91 (95%CI: 0.71–1.17), P: 0.469), a negative history of hypertension (positive vs negative: 1.03 (95%CI: 0.92–1.14), P: 0.648), a negative history of diabetes (positive vs negative: 1.25 (95%CI: 1.07–1.47), P: 0.005), the absence of abdominal obesity (positive vs negative: 1.1 (95%CI: 0.98–1.24), P: 0.106), hypercholesterolemia (positive vs negative: 0.94 (95%CI: 0.84–1.04), P: 0.236), and a history of CVD (positive vs negative: 1.21 (95%CI: 0.86–1.71), P: 0.275). Upon comparing genders, the adjusted odds ratio of IPA was generally higher in women of higher socioeconomic status and men of lower socioeconomic status. Moreover, high serum cholesterol levels were associated with lower odds of IPA in females (0.86 (95%CI: 0.75–0.99), P: 0.039), whereas this was not the case among male subjects (1.06 (95%CI: 0.89–1.26), P: 0.504). Other association patterns regarding males and females either remained the same or differed slightly.

**Table 3:** Gender-specific association of sociodemographic, life style characteristics, and metabolic risk factors with insufficient physical activity among Iranian population

Variable	Categories	Total			Male			Female		
		Crude OR (95% CI)	Adjusted OR (95% CI)^a^	Fully Adjusted OR (95% CI)^b^	Crude OR (95% CI)	Adjusted OR (95% CI)^a^	Fully Adjusted OR (95% CI)^b^	Crude OR (95% CI)	Adjusted OR (95% CI)^a^	Fully Adjusted OR (95% CI)^b^
Sociodemographic Characteristics										
Gender	Female	1·0	1·0	1·0	–	–	–	–	–	–
	Male	0·51 (0·48–0·54), P:< 0·001	0·62 (0·56–0·69), P:< 0·001	0·69 (0·58–0·82), P:< 0·001	–	–	–	–	–	–
Age	18–24 years	1·0	1·0	–	1·0	1·0	–	1·0	1·0	–
	25–34 years	1·15 (1·04–1·26), P:0·006	1·03 (0·92–1·16), P:0·579	1·0	1·31 (1·12–1·53), P:0·001	1·23 (1–1·51), P:0·048	1·0	1·06 (0·93–1·21), P:0·386	0·96 (0·83–1·12), P:0·635	1·0
	35–44 years	1·2 (1·09–1·32), P:< 0·001	1·03 (0·91–1·17), P:0·636	0·99 (0·88–1·12), P:0·855	1·77 (1·51–2·07), P:< 0·001	1·58 (1·26–1·99), P:< 0·001	1·09 (0·9–1·33), P:0·37	0·92 (0·8–1·05), P:0·213	0·82 (0·7–0·96), P:0·013	0·92 (0·78–1·07), P:0·27
	45–54 years	1·12 (1·01–1·24), P:0·028	0·9 (0·79–1·03), P:0·121	0·78 (0·68–0·89), P:< 0·001	1·78 (1·52–2·09), P:< 0·001	1·49 (1·18–1·89), P:0·001	0·91 (0·73–1·13), P:0·406	0·81 (0·7–0·92), P:0·002	0·67 (0·57–0·79), P:< 0·001	0·7 (0·59–0·84), P:< 0·001
	55–64 years	1·13 (1·01–1·25), P:0·026	0·89 (0·77–1·02), P:0·098	0·76 (0·64–0·89), P:0·001	1·58 (1·34–1·86), P:< 0·001	1·2 (0·93–1·53), P:0·154	0·74 (0·57–0·95), P:0·018	0·92 (0·8–1·06), P:0·232	0·76 (0·64–0·91), P:0·002	0·78 (0·63–0·97), P:0·027
Residence area	Rural	1·0	1·0	1·0	1·0	1·0	1·0	1·0	1·0	1·0
	Urban	1·48 (1·39–1·57), P:< 0·001	1·61 (1·49–1·73), P:< 0·001	1·69 (1·52–1·88), P:< 0·001	1·51 (1·37–1·66), P:< 0·001	1·61 (1·42–1·81), P:< 0·001	1·76 (1·49–2·09), P:< 0·001	1·56 (1·44–1·69), P:< 0·001	1·63 (1·48–1·79), P:< 0·001	1·66 (1·45–1·9), P:< 0·001
Marital status	Never married	1·0	1·0	1·0	1·0	1·0	1·0	1·0	1·0	1·0
	Married	1·33 (1·24–1·43), P:< 0·001	1·28 (1·16–1·41), P:< 0·001	1·11 (0·94–1·3), P:0·219	1·42 (1·28–1·58), P:< 0·001	1·24 (1·06–1·46), P:0·007	1·25 (0·97–1·63), P:0·088	1·1 (0·99–1·22), P:0·07	1·1 (0·96–1·26), P:0·154	0·95 (0·77–1·19), P:0·673
	Divorced or Living alone	1·6 (1·31–1·95), P:< 0·001	1·33 (1·07–1·66), P:0·011	1·37 (0·96–1·96), P:0·079	1·77 (1·15–2·7), P:0·009	1·58 (0·99–2·52), P:0·055	1·61 (0·76–3·42), P:0·212	1·09 (0·86–1·38), P:0·479	1·16 (0·9–1·5), P:0·254	1·25 (0·83–1·89), P:0·283
	Widow	1·64 (1·4–1·93), P:< 0·001	1·25 (1·04–1·51), P:0·017	1·09 (0·83–1·45), P:0·529	1·95 (0·95–4·01), P:0·07	1·57 (0·71–3·46), P:0·262	1·14 (0·42–3·1), P:0·792	1·04 (0·87–1·24), P:0·654	1·13 (0·91–1·39), P:0·26	0·95 (0·69–1·3), P:0·743
Occupation	Nonpaid & self-paid	1·0	1·0	1·0	1·0	1·0	1·0	1·0	1·0	1·0
	Employee	1·42 (1·26–1·59), P:< 0·001	1·25 (1·1–1·43), P:0·001	1·1 (0·91–1·34), P:0·326	1·24 (1·08–1·42), P:0·002	1·21 (1·03–1·41), P:0·017	1·01 (0·8–1·28), P:0·94	1·61 (1·25–2·09), P:< 0·001	1·55 (1·17–2·05), P:0·002	1·46 (0·95–2·24), P:0·086
	Worker	1·25 (1·09–1·45), P:0·002	1·08 (0·93–1·26), P:0·324	0·92 (0·72–1·17), P:0·503	1·19 (1–1·41), P:0·045	1·16 (0·96–1·38), P:0·118	1·04 (0·79–1·38), P:0·762	1·13 (0·85–1·5), P:0·401	1·03 (0·76–1·39), P:0·855	0·69 (0·42–1·16), P:0·161
	Student	1·17 (1·02–1·33), P:0·022	1·08 (0·92–1·28), P:0·344	0·96 (0·63–1·47), P:0·853	0·72 (0·59–0·86), P:< 0·001	1·11 (0·87–1·43), P:0·396	0·79 (0·4–1·56), P:0·503	1·47 (1·15–1·87), P:0·002	1·27 (0·96–1·68), P:0·094	1·15 (0·6–2·19), P:0·675
	Soldier	0·79 (0·48–1·3), P:0·357	0·95 (0·57–1·58), P:0·838	1·03 (0·29–3·66), P:0·969	0·82 (0·5–1·35), P:0·432	1·19 (0·71–2), P:0·514	1·28 (0·35–4·64), P:0·705	–	–	–
	Unemployed & retired	1·41 (1·28–1·56), P:< 0·001	1·38 (1·24–1·54), P:< 0·001	1·3 (1·11–1·53), P:0·002	1·32 (1·19–1·48), P:< 0·001	1·4 (1·23–1·58), P:< 0·001	1·39 (1·15–1·67), P:0·001	1·45 (1·14–1·85), P:0·002	1·47 (1·14–1·9), P:0·003	1·16 (0·76–1·77), P:0·497
	Housekeeper	2·11 (1·96–2·26), P:< 0·001	1·38 (1·23–1·54), P:< 0·001	1·35 (1·12–1·63), P:0·001	2·67 (1·73–4·11), P:< 0·001	2·57 (1·64–4·05), P:< 0·001	2·55 (1·4–4·65), P:0·002	1·59 (1·33–1·89), P:< 0·001	1·67 (1·39–2·01), P:< 0·001	1·4 (1·05–1·88), P:0·024
Education	Illiterate	1·0	1·0	1·0		1·0	1·0		1·0	1·0
	1–6 years	0·93 (0·84–1·03), P:0·172	0·93 (0·83–1·04), P:0·198	0·91 (0·79–1·06), P:0·226	0·87 (0·71–1·06), P:0·165	0·77 (0·62–0·95), P:0·016	0·74 (0·56–0·99), P:0·044	1·09 (0·97–1·23), P:0·166	0·96 (0·84–1·09), P:0·514	0·96 (0·81–1·13), P:0·599
	7–12 years	0·98 (0·89–1·08), P:0·654	1·02 (0·91–1·14), P:0·745	0·97 (0·83–1·14), P:0·717	0·96 (0·8–1·16), P:0·668	0·91 (0·74–1·13), P:0·405	0·86 (0·64–1·15), P:0·308	1·24 (1·1–1·38), P:< 0·001	0·96 (0·84–1·11), P:0·608	0·95 (0·78–1·16), P:0·631
	> 12 years	0·88 (0·79–0·97), P:0·013	0·94 (0·82–1·08), P:0·37	1·02 (0·84–1·24), P:0·857	0·84 (0·69–1·02), P:0·08	0·76 (0·6–0·96), P:0·022	0·81 (0·57–1·14), P:0·228	1·16 (1·02–1·32), P:0·022	0·99 (0·84–1·17), P:0·924	1·1 (0·86–1·4), P:0·461
Wealth index	1st quintile (poorest)	1·0	1·0	1·0	1·0	1·0	1·0	1·0	1·0	1·0
	2nd quintile	1·19 (1·09–1·31), P:< 0·001	1·03 (0·94–1·14), P:0·511	1·1 (0·96–1·27), P:0·172	1·06 (0·92–1·23), P:0·394	0·92 (0·78–1·08), P:0·282	0·95 (0·75–1·21), P:0·668	1·32 (1·17–1·48), P:< 0·001	1·11 (0·98–1·26), P:0·088	1·22 (1·02–1·45), P:0·031
	3rd quintile	1·18 (1·08–1·28), P:< 0·001	0·96 (0·87–1·06), P:0·465	0·95 (0·82–1·09), P:0·453	1·01 (0·88–1·16), P:0·9	0·77 (0·65–0·9), P:0·001	0·71 (0·56–0·9), P:0·004	1·38 (1·23–1·55), P:< 0·001	1·14 (1–1·29), P:0·046	1·12 (0·93–1·35), P:0·247
	4th quintile	1·33 (1·22–1·45), P:< 0·001	1·06 (0·96–1·17), P:0·251	1·06 (0·91–1·25), P:0·433	1·17 (1·02–1·33), P:0·023	0·88 (0·75–1·04), P:0·125	0·88 (0·69–1·13), P:0·331	1·58 (1·41–1·77), P:< 0·001	1·22 (1·07–1·39), P:0·003	1·21 (0·99–1·49), P:0·061
	5th quintile (richest)	1·13 (1·04–1·22), P:0·006	0·87 (0·79–0·97), P:0·011	0·88 (0·74–1·04), P:0·137	1·09 (0·96–1·24), P:0·179	0·78 (0·67–0·92), P:0·003	0·73 (0·56–0·95), P:0·02	1·24 (1·11–1·39), P:< 0·001	0·96 (0·83–1·1), P:0·526	0·99 (0·79–1·25), P:0·942
Life style related variables										
Appropriate fruit and vegetable consumption	No	1·35 (1·24–1·47), P:< 0·001	1·43 (1·26–1·63), P:< 0·001	1·4 (1·23–1·59), P:< 0·001	1·41 (1·24–1·6)), P:< 0·001	1·58 (1·31–1·91), P:< 0·001	1·62 (1·32–1·99), P:< 0·001	1·27 (1·13–1·43), P:< 0·001	1·29 (1·09–1·53), P:0·003	1·28 (1·07–1·52), P:0·006
	Yes	1·0	1·0	1·0	1·0	1·0	1·0	1·0	1·0	1·0
High salt intake	No	1·0	1·0	1·0	1·0	1·0	1·0	1·0	1·0	1·0
	Yes	1·22 (1·05–1·42), P:0·011	1·21 (1·04–1·41), P:0·014	1·06 (0·9–1·24), P:0·5	1·2 (0·88–1·65), P:0·242	1·21 (0·88–1·68), P:0·242	1·16 (0·82–1·63), P:0·402	1·02 (0·85–1·21), P:0·854	1·02 (0·85–1·21), P:0·86	1·05 (0·88–1·26), P:0·573
Smoking	Never smoker	1·0	1·0	1·0	1·0	1·0	1·0	1·0	1·0	1·0
	Ever smoker	0·78 (0·72–0·84), P:< 0·001	0·85 (0·75–0·96), P:0·008	1·24 (1·07–1·43), P:0·004	1·2 (1·09–1·31), P:< 0·001	1·18 (1·03–1·35), P:0·018	1·2 (1·03–1·4), P:0·019	0·92 (0·68–1·25), P:0·602	1·01 (0·65–1·57), P:0·965	1·08 (0·69–1·68), P:0·733
Alcohol consumption	No	1·0	1·0	1·0	1·0	1·0	1·0	1·0	1·0	1·0
	Yes	0·62 (0·52–0·75), P:< 0·001	0·65 (0·55–0·76), P:< 0·001	0·78 (0·65–0·93), P:0·005	0·87 (0·71–1·07), P:0·187	0·81 (0·68–0·96), P:0·015	0·87 (0·72–1·04), P:0·135	0·66 (0·38–1·14), P:0·138	0·57 (0·34–0·96), P:0·036	0·48 (0·29–0·8), P:0·005
Injury	No	1·0	1·0	1·0	1·0	1·0	1·0	1·0	1·0	1·0
	Yes	0·9 (0·82–1), P:0·041	0·96 (0·83–1·1), P:0·562	0·99 (0·85–1·14), P:0·861	0·94 (0·81–1·08), P:0·359	0·94 (0·77–1·15), P:0·551	1 (0·8–1·25), P:0·999	0·94 (0·82–1·07), P:0·335	0·98 (0·81–1·2), P:0·872	1 (0·82–1·22), P:0·999
Personal car ownership	No	1·0	1·0	1·0	1·0	1·0	1·0	1·0	1·0	1·0
	Yes	1·09 (1·03–1·15), P:0·002	1·18 (1·09–1·28), P:< 0·001	1·23 (1·11–1·35), P:< 0·001	1·2 (1·11–1·31), P:< 0·001	1·36 (1·2–1·54), P:< 0·001	1·37 (1·17–1·6), P:< 0·001	1·12 (1·04–1·2), P:0·003	1·19 (1·07–1·33), P:0·001	1·14 (1–1·3), P:0·051
Metabolic risk factors										
Hypertension	No	1·0	1·0	1·0	1·0	1·0	1·0	1·0	1·0	1·0
	Yes	1·06 (0·99–1·13), P:0·079	0·91 (0·83–1·01), P:0·075	1·03 (0·92–1·14), P:0·648	1·15 (1·04–1·27), P:0·008	1·02 (0·87–1·18), P:0·829	1·09 (0·92–1·29), P:0·319	0·99 (0·9–1·08), P:0·771	0·89 (0·78–1·02), P:0·09	0·97 (0·84–1·12), P:0·686
Past medical history of cardiovascular disease	No	1·0	1·0	1·0		1·0	1·0		1·0	1·0
	Yes	1·51 (1·09–2·09), P:0·013	1·09 (0·79–1·51), P:0·608	1·21 (0·86–1·71), P:0·275	1·93 (1·26–2·95), P:0·002	1·28 (0·86–1·9), P:0·222	1·28 (0·84–1·94), P:0·245	1·32 (0·8–2·18), P:0·283	1·04 (0·59–1·85), P:0·887	1·04 (0·57–1·9), P:0·892
Diabetes mellitus	No	1·0	1·0	1·0	1·0	1·0	1·0	1·0	1·0	1·0
	Yes	1·23 (1·06–1·42), P:0·005	1·19 (1·02–1·38), P:0·025	1·25 (1·07–1·47), P:0·005	1·17 (0·94–1·46), P:0·152	1·04 (0·83–1·3), P:0·752	1·07 (0·83–1·37), P:0·606	1·26 (1·05–1·53), P:0·015	1·36 (1·11–1·67), P:0·003	1·39 (1·12–1·71), P:0·002
Dyslipidemia	No	1·0	1·0	1·0	1·0	1·0	1·0	1·0	1·0	1·0
	Yes	1 (0·9–1·1), P:0·926	0·91 (0·82–1), P:0·057	0·94 (0·84–1·04), P:0·236	1·15 (0·99–1·34), P:0·064	1·07 (0·91–1·25), P:0·412	1·06 (0·89–1·26), P:0·504	0·87 (0·77–0·99), P:0·03	0·82 (0·72–0·94), P:0·004	0·86 (0·75–0·99), P:0·039
Abdominal obesity	No	1·0	1·0	1·0	1·0	1·0	1·0	1·0	1·0	1·0
	Yes	1·42 (1·34–1·5), P:< 0·001	1·39 (1·25–1·54), P:< 0·001	1·1 (0·98–1·24), P:0·106	1·45 (1·31–1·61), P:< 0·001	1·27 (1·06–1·53), P:0·009	1·25 (1·03–1·52), P:0·027	1·01 (0·93–1·08), P:0·864	0·99 (0·85–1·14), P:0·848	1·04 (0·9–1·21), P:0·611
BMI (body mass index)	Under weight	1·0	1·0	1·0	1·0	1·0	1·0	1·0	1·0	1·0
	Normal weight	1·07 (0·93–1·24), P:0·334	0·86 (0·68–1·08), P:0·191	0·91 (0·71–1·17), P:0·469	1·17 (0·95–1·44), P:0·142	0·95 (0·69–1·3), P:0·736	0·99 (0·7–1·39), P:0·946	0·95 (0·77–1·16), P:0·597	0·75 (0·53–1·05), P:0·097	0·84 (0·58–1·21), P:0·339
	Over weight	1·09 (1·02–1·16), P:0·008	0·95 (0·85–1·05), P:0·304	0·96 (0·86–1·07), P:0·464	1·16 (1·06–1·28), P:0·001	0·99 (0·86–1·15), P:0·913	0·98 (0·83–1·15), P:0·775	0·98 (0·9–1·08), P:0·717	1·02 (0·88–1·19), P:0·753	0·96 (0·82–1·11), P:0·565
	Obesity	1·38 (1·28–1·48), P:< 0·001	1·03 (0·9–1·17), P:0·7	1·06 (0·92–1·22), P:0·44	1·47 (1·31–1·66), P:< 0·001	1·13 (0·91–1·41), P:0·271	1·14 (0·89–1·44), P:0·295	1·08 (0·98–1·19), P:0·106	1·1 (0·93–1·32), P:0·271	1·03 (0·86–1·24), P:0·759

*OR* Odds Ratio, *95% CI* 95% Confidence Interval

^a^and^b^ report ORs separately and concurrently adjusted for sociodemographic, life style related variables, and metabolic risk factors, respectively

### Provinces

**Fig. 4:**
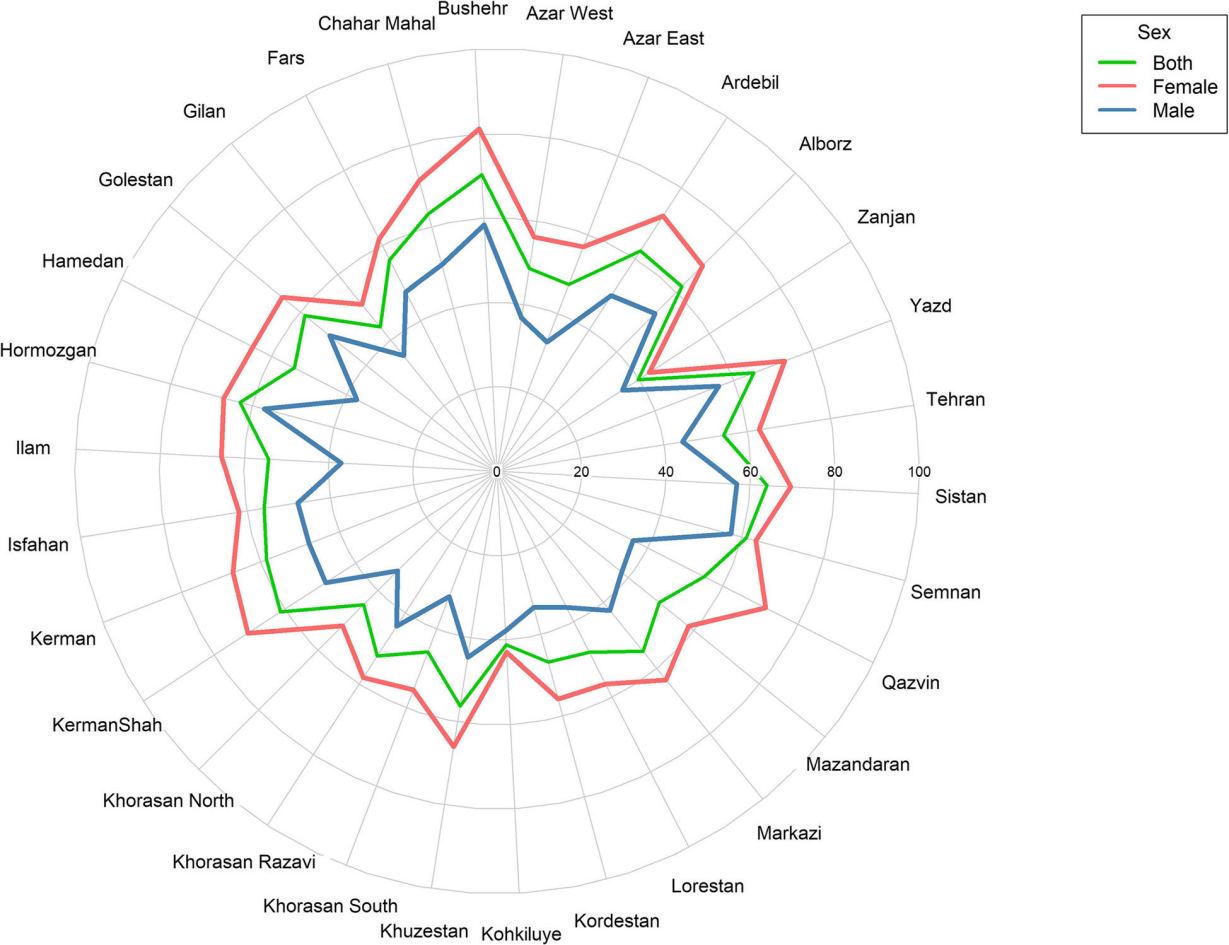
Prevalence of insufficient physical activity in the male and female adult populations across Iran’s provinces, excluding Qom

The highest IPA prevalence was observed in Boushehr, with a rate of 81.1% among females (95% CI: 75.5–86.7), 58.3% among males (95% CI: 50.1–66.6) and an overall rate of 70.3% (95% CI: 65.1–75.5), respectively (Fig. 4). The lowest overall, male, and female IPA prevalence rates were observed in Zanjan (39.8%, 95% CI: 35.6–44.0), South Khorasan (31.8%, 95% CI: 25.0–38.6), and Zanjan (42.9%, 95% CI: 37.4–48.4). At the provincial level, there was a non-significant trend of higher IPA in the more urbanized provinces with a correlation *r* = 0.24 and *P* value = 0.20. However, provinces with a higher than average wealth index had a significantly higher prevalence of IPA (*r* = − 0.68, *P* value< 0.001).

## Discussion

We found that, in 2016, more than half the Iranian adult population (54.7%) had insufficient physical activity (IPA), less than the minimum suggested physical activity recommended by the WHO [22]. Due to different methodologies applied mainly in sampling, a careful comparison showed that it increased from 39.1% in 2011 [14], 33.5% in 2010 (WHO report) [22], and 40.0% in 2007 [12]. However, the 2005 STEPS study reported higher IPA (68.8%) without detailing the methods of PA assessment [17]. Different methodologies were the result of different protocols, mainly in sampling and the questionnaires (GPAQ vs. International physical activity questionnaire or IPAQ) used as instruments. Our investigated prevalence of IPA was two times greater than the global IPA (25% based on WHO’s last report), nearly 1.5 times greater than the Eastern Mediterranean Region (31%) and two times greater than the upper-middle income countries (25.4%) [22].

When comparing the Iranian IPA rate with other middle-income countries, the only country with a higher IPA was Colombia (63.6%). In the Eastern Mediterranean Region (EMR), the Iranian IPA rate was greater than all other countries except Kuwait (56.6) [22]. The difference between female (61.9%) and male (45.3%) IPA rate in our country was double, triple, and nearly 1.5 times of the difference globally (males: 20%; females: 27%), in middle-income countries (males: 22.8%; females: 28.1%), and in the EMR (males: 25.6%; females: 37.1%), respectively [22]. There are a few countries, economically or geographically similar to Iran, with higher inter-gender differences in IPA rates; Bahamas, Colombia, Dominica, Gabon, Saint Lucia, and Tonga [22]. Colombia, Kuwait, and Saudi Arabia had higher female IPA rates compared to Iranian females, and Colombia, Iraq, Kuwait, Malaysia, and Saudi Arabia’s male IPA rates were higher than those of Iranian males [22]. Being female noticeably increased the odds of IPA [OR male vs. female 0.69 (0.58–0.82)] in Iran.

Gender disparity is a well-known observation around the world which is at its peak in low and middle-income countries (LMIC) [24, 25], and has repeatedly been observed in other studies of LMIC with an OR of 1.3 to 1.7 [26, 27]. The human development index (HDI) has been known to correlate with the prevalence of physical inactivity worldwide, and generally speaking, an increase in the HDI is associated with an increased IPA [28]. One of the components of HDI is gender inequality and although Iran ranks 68th in HDI globally, it ranks 118th in the Gender Inequality Index (GII) [29]. Iran has one of the highest gender differences in adult IPA worldwide [30]. The high GII is indicative of possible barriers for the female population to participate in social activities that involve physical activity [29]. In recent years, in HIC (high-income countries) that mainly have high HDIs, the recreational domain of physical activity has increased concurrent to reduced PA at work, a similar finding among young Iranian males, not females [25].

Upon assessing the correlates of IPA in the Iranian population, the general pattern was similar to that in other LMIC [25]; being female, an urban resident, and having a blue-collar occupation. Unhealthy dietary habits, daily smoking and history of diabetes were correlates of increased IPA odds in our population. This finding underscores the significance of the joint occurrence of IPA and CVD risks which exaggerates the attributable risk of IPA. Meanwhile, it gives the notion that policies against IPA would be even more beneficial in populations who already have other CVD risk factors. Moreover, as age increased, congruent with previous studies in Iran [12, 17], the prevalence of absolute inactivity increased and the total calculated METs of the population decreased; however, the adjusted OR of IPA presented a decreasing pattern. This suggests that the age-related increase in IPA might be manipulated by lifestyle - related factors other than the aging process. These include, a change in the occupational status, marriage, and possession of a personal vehicle. This finding is consistent with earlier findings of sociological theories; loneliness, as expected, increased the odds of IPA [31], and possession of a vehicle increased the IPA regardless of socioeconomic status [32]. Finally, the impact of occupation on IPA is greater than the direct PA demonstrated during worktime, as social networks and role-related demands affected by occupation are drivers of PA [33]. Alcohol consumption was associated with lower IPA adjusted-odds, congruent with previous international reports, but against health experts’ hypotheses [34, 35]. Risk-taking characteristics, the social nature of PA and alcohol consumption, being physically active for cosmetic purposes in alcohol users, and finally, peer influence in young-age might have resulted in this association [34, 35].

Despite having higher IPA, there was less sedentary lifestyle among the Iranian population than observed globally (41.5%) and in the EMR (41.4%) [25]. Similar to our findings, the age pattern of sedentary lifestyle demonstrated a U-shaped pattern [25]. As shown before, sedentary time increased with a combination of female gender and urbanization [36], which emphasizes the need for actions against IPA to eliminate or at least lessen the associated risks of sedentary lifestyle.

The higher prevalence of IPA compared to geographically and economically similar countries, the vicious cycle between NCDs and IPA, the high population IPA attributable fraction of dementia in Iran [25], the higher burden of IPA in developing countries [15], and finally, reports of effective interventions on PA promotion [37, 38] make IPA a vital and feasible-to-improve epidemiological issue in Iran. Moreover, earlier policies seem to be inefficiently or insufficiently planned and implemented. For instance, the Ministry of Health and Medical Education introduced the National Physical Activity Plan for Health Promotion in Iran in the year 2016 [39]. However, there has since been no report on the implementation or evaluation of the program. In addition, there are no valid or official reports on any previous plans or programs in Iran that targeted physical activity [39]. The absence of such official reports indicates the necessity of examining and updating the status of physical inactivity. For an effective and cost-efficient action plan, policies should be targeted towards specific subgroups of populations, as most of the IPA attributes are demographic and biologic in LMIC countries [40], similar to Iran. However, although gender specific policies may decrease IPA more effectively, they may exacerbate the present gender gap [29]. Importantly, before any budget - demanding actions, pilot studies should be conducted to specify potentially effective domains for different age and socio-demographic categories of the population. The total IPA prevalence itself, does not vary much between age categories, however, when stratifying results into genders and PA domains, females in young age groups, and the transport domain in the middle-aged group, are possible targets for age-specific interventions. Work domain inactivity, despite its significant role in IPA, might not be easy to change, highlighting active transport and recreational domains of physical activity as potential fields, both of which have been influenced by urbanization and cultural boundaries. A PA-friendly environment can make up for half the weekly needed PA in urban areas [41] and reduce the impact of using a personal vehicle on IPA [41] –demonstrated as a key associate of IPA in our population. Furthermore, recreation, a PA domain largely responsible for the inter-gender difference in our study population, may include vigorous intensity of PA, shown to be highly time-efficient against NCD risks [42]. This domain of physical activity is of great potential and a target to be addressed in further policies of the country. Moreover, previously planned policies and programs that have intended to increase recreational physical activity have not succeeded [12, 14, 17, 22]. These programs mainly consisted of inadequately calibrated PA-friendly microenvironments in public parks in limited cities of Iran, that have not been accompanied by sufficient and effective education [43]. These types of interventions have proven to be effective in many HIC as well as few documentations in LMIC [25].

The present study is the largest national survey of PA in Iran, including samples from all its provinces except one, representative of its population characteristics. The design, sampling and implementation of the study were in accordance with WHO’s STEPS methods and the data collection process was performed entirely online. Since all subjects were sampled in spring and summer, our study has not been affected by seasonal changes in PA. However, lack of data from one province may have partially influenced the representativeness of our sample. We tried to address this issue by weighted statistical analysis of data from other provinces to mimic Iran’s population in 2016. Second, the self-reported PA data (using the GPAQ questionnaire) may be subject to recall bias, over-reporting due to social preferences and potential biases during translation of the questionnaire. Moreover, the validity and reliability may be different in different regions, especially in rural and urban areas. As a recently proposed key factor in PA [41], physical environment has not been assessed in the present study. The focus of the present study was on the adult population, while it has been shown that IPA is even more prevalent among children and adolescents [25, 44]. Finally, the different methodologies that have been employed in previous national surveys should be addressed to enable policymakers to understand the trends and statistical points regarding IPA, independent of complex statistical analyses.

## Conclusion

Taken together, our results are indicative of substantial IPA among the Iranian population, which, with a less sensitive recommended cut-point by WHO in recent years, has increased in comparison with recent previous studies. The gender gap in IPA is high and genders have grown more apart. Accelerated urbanization and lifestyle changes concurrent to high IPA frequency, underscore the future health consequences of this global issue. The association of unhealthy dietary habits and diabetes as well as IPA in our population highlights the absolute need for interventions increasing PA. A prompt initiative for population-specific actions should be taken to address different aspects of PA in Iran, especially among the female population.

## Supplementary information

**Additional file 1:** Protocol Design for Large–Scale Cross–Sectional Studies on Surveillance of Non–Communicable Diseases Risk Factors in Iran: STEPS 2016. Protocol of the 2016 STEPS study, the main project from which the current study has originated from. (DOCX 384 kb)

## Data Availability

The datasets used and/or analyzed during the current study are available upon reasonable request from the corresponding author and aggregated reports are published online on vizit.ncdrc.info. The aggregated data and visualizations on visit platform are freely available to public for non-commercial use.

## Abbreviations

ALT: Alanine Aminotransferase
BMI: Body mass index
CVD: Cardiovascular diseases
GPAQ: Global Physical Activity Questionnaire
HDI: Human development index
IPA: Insufficient physical activity
LMIC: Low and middle-income country
MET: Metabolic equivalent of task
NCDs: Non-communicable diseases
PA: Physical activity
STEPS: STEPwise approach to risk factor Surveillance
