# Supplementary material for: Physical activity profile of the Iranian population: STEPS survey, 2016
Source: BMC Public Health. 2019 Sep 13;19:1266. doi: 10.1186/s12889-019-7592-5 (PMC6743153; doi:10.1186/s12889-019-7592-5)
Supplement: Supplementary file 1 — Additional file 1: Protocol Design for Large–Scale Cross–Sectional Studies on Surveillance of Non–Communicable Diseases Risk Factors in Iran: STEPS 2016. Protocol of the 2016 STEPS study, the main project from which the current study has originated from. (DOCX 384 kb) [file 12889_2019_7592_MOESM1_ESM.docx]

Table S1. Frequency and association of insufficient physical activity among Iranian population according to the sociodemographic, lifestyle, anthropometry and laboratory measurements variables

| **Variables** | **Categories** | **Prevalence Total** | **Prevalence Male** | **Prevalence Female** |
| --- | --- | --- | --- | --- |
|  |  | (95% CI) | (95% CI) | (95% CI) |
| **Total** | | 54.6 % (54.0-55.3) | 45.3 % (44.3-46.3) | 61.9% (61.0-62.7) |
| **Age** | 18-24 years | 51.6% (49.6-53.6) | 63.4% (60.8-65.9) | 35.4% (32.4-38.5) |
|  | 25-34 years | 55% (53.7-56.3) | 64.7% (63.1-66.3) | 41.8% (39.8-43.7) |
|  | 35-44 years | 56.1% (54.7-57.4) | 61.4% (59.7-63.1) | 49.3% (47.2-51.3) |
|  | 45-54 years | 54.4% (53-55.8) | 58.2% (56.4-60.1) | 49.4% (47.2-51.6) |
|  | 55-64 years | 54.5% (52.9-56.1) | 61.3% (59.2-63.4) | 46.4% (44-48.8) |
| **Residence area** | Rural | 47.5% (46.2-48.8) | 37.7% (35.8-39.7) | 54.1% (52.5-55.8) |
|  | Urban | 57.2% (56.5-58) | 47.8% (46.6-48.9) | 64.8% (63.9-65.8) |
| **Marital status** | Never married | 48.5% (46.9-50.2) | 38.4% (36.1-40.7) | 60% (57.7-62.3) |
|  | Married | 55.6% (54.9-56.4) | 47% (45.9-48.1) | 62.3% (61.4-63.3) |
|  | Divorced or Living alone | 60.1% (55.5-64.7) | 52.4% (42.1-62.7) | 62% (57-67.1) |
|  | Widow | 60.7% (57.2-64.2) | 54.8% (37.1-72.6) | 61% (57.4-64.5) |
| **Occupation** | Nonpaid & self-paid | 44.6% (43.2-46) | 43.7% (42.3-45.2) | 51.6% (47.3-55.8) |
|  | Employee | 53.3% (50.7-55.8) | 49.1% (46.1-52.1) | 63.2% (58.7-67.7) |
|  | Worker | 50.2% (47-53.5) | 48.1% (44.1-52.1) | 54.6% (48.9-60.3) |
|  | Student | 48.4% (45.4-51.4) | 35.8% (31.7-39.8) | 61% (56.9-65) |
|  | Soldier | 38.9% (27.2-50.6) | 38.9% (27.2-50.6) | - |
|  | Unemployed & retired | 53.2% (51.2-55.3) | 50.7% (48.4-53.1) | 60.7% (56.7-64.8) |
|  | Housekeeper | 62.9% (61.9-63.8) | 67.5% (58.1-76.9) | 62.8% (61.9-63.8) |
| **Education** | Illiterate | 56.0% (53.9-58.1) | 47.6% (43.2-52.0) | 58.5% (56.2-60.9) |
|  | 1-6 years | 54.3% (52.9-55.6) | 44.1% (41.9-46.3) | 60.6% (58.9-62.2) |
|  | 7-12 years | 55.5% (54.5-56.5) | 46.6% (45.1-48.0) | 63.5% (62.2-64.9) |
|  | 12< years | 52.8% (51.4-54.2) | 43.3% (41.3-45.2) | 62.1% (60.2-64.0) |
| **Wealth index** | 1^st^ quintile (poorest) | 51% (49.4-52.5) | 43.7% (41.3-46.2) | 55.8% (53.8-57.8) |
|  | 2^nd^ quintile | 55.4% (53.8-57) | 45.2% (42.8-47.7) | 62.4% (60.4-64.4) |
|  | 3^rd^ quintile | 55% (53.5-56.5) | 43.9% (41.6-46.3) | 63.5% (61.6-65.4) |
|  | 4^th^ quintile | 58.1% (56.6-59.5) | 47.6% (45.4-49.7) | 66.6% (64.7-68.4) |
|  | 5^th^ quintile (richest) | 53.9% (52.5-55.3) | 45.9% (43.9-48) | 61.1% (59.2-63) |
| **Appropriate fruit and vegetable consumption** | No | 55.5% (54.8-56.2) | 46.3% (45.2-47.3) | 62.5% (61.6-63.3) |
|  | Yes | 48% (46.1-50) | 38% (35.1-40.8) | 56.7% (54-59.3) |
| **High salt intake** | No | 54.6% (53.6-55.6) | 46.4% (44.9-47.9) | 60.8% (59.5-62.1) |
|  | Yes | 59.4% (55.9-63) | 51.1% (43.4-58.7) | 61.2% (57.2-65.1) |
| **Ever daily cigarette smoking** | No | 55.5% (54.8-56.2) | 44.1% (42.9-45.3) | 61.9% (61-62.7) |
|  | Yes | 49.2% (47.4-51.1) | 48.5% (46.6-50.5) | 59.9% (52.5-67.3) |
| **Alcohol consumption** | No | 54.8% (54.2-55.5) | 45.3% (44.3-46.4) | 61.8% (61-62.7) |
|  | Yes | 43% (38.5-47.5) | 42% (37.2-46.8) | 51.5% (37.6-65.4) |
| **Injury** | No | 54.9% (54.2-55.6) | 45.5% (44.4-46.5) | 62% (61.1-62.9) |
|  | Yes | 52.4% (50.1-54.7) | 43.8% (40.5-47.1) | 60.4% (57.3-63.5) |
| **Personal car ownership** | No | 53.6% (52.7-54.6) | 42.8% (41.2-44.4) | 60.6% (59.4-61.8) |
|  | Yes | 55.7% (54.8-56.7) | 47.4% (46-48.7) | 63.3% (62-64.5) |
| **Hypertension** | No | 54.4% (53.7-55.2) | 44.6% (43.5-45.7) | 62% (61-63) |
|  | Yes | 55.9% (54.4-57.4) | 48.1% (45.8-50.4) | 61.7% (59.8-63.6) |
| **Past medical history of cardiovascular disease** | No | 56.7% (55.7-57.6) | 47.9% (46.4-49.4) | 63.5% (62.2-64.7) |
|  | Yes | 66.4% (59.2-73.5) | 63.9% (54.2-73.6) | 69.6% (59-80.2) |
| **Diabetes mellitus** | No | 55.0% (54.0-56.0) | 46.5% (44.9-48.0) | 60.8% (59.5-62.1) |
|  | Yes | 60.0% (56.7-63.2) | 50.5% (45.2-55.7) | 66.2% (62.1-70.2) |
| **Dyslipidemia** | No | 55.5% (54.4-56.6) | 46.2% (44.5-47.8) | 62% (60.7-63.4) |
|  | Yes | 55.4% (53.3-57.5) | 49.7% (46.3-53.1) | 58.7% (56.1-61.4) |
| **Abdominal obesity** | No | 50.8% (49.9-51.6) | 43.2% (42.1-44.3) | 61.3% (60-62.7) |
|  | Yes | 59.4% (58.4-60.5) | 52.5% (50.3-54.8) | 61.5% (60.3-62.7) |
| **BMI (body mass index)** | Under weight | 53.3% (49.9-56.7) | 45.8% (40.9-50.8) | 59.8% (55.2-64.3) |
|  | Normal weight | 51.5% (50.4-52.6) | 42% (40.3-43.6) | 61.1% (59.5-62.6) |
|  | Over weight | 53.7% (52.5-54.8) | 45.7% (44.1-47.3) | 60.7% (59.2-62.1) |
|  | Obesity | 59.4% (58-60.7) | 51.6% (49.1-54.1) | 62.9% (61.3-64.5) |

OR: Odds Ratio, 95% CI: 95% Confidence Interval

*** = *p*-value < 0.001

** = *p*-value < 0.01

* = *p*-value < 0.05

Additional figures:


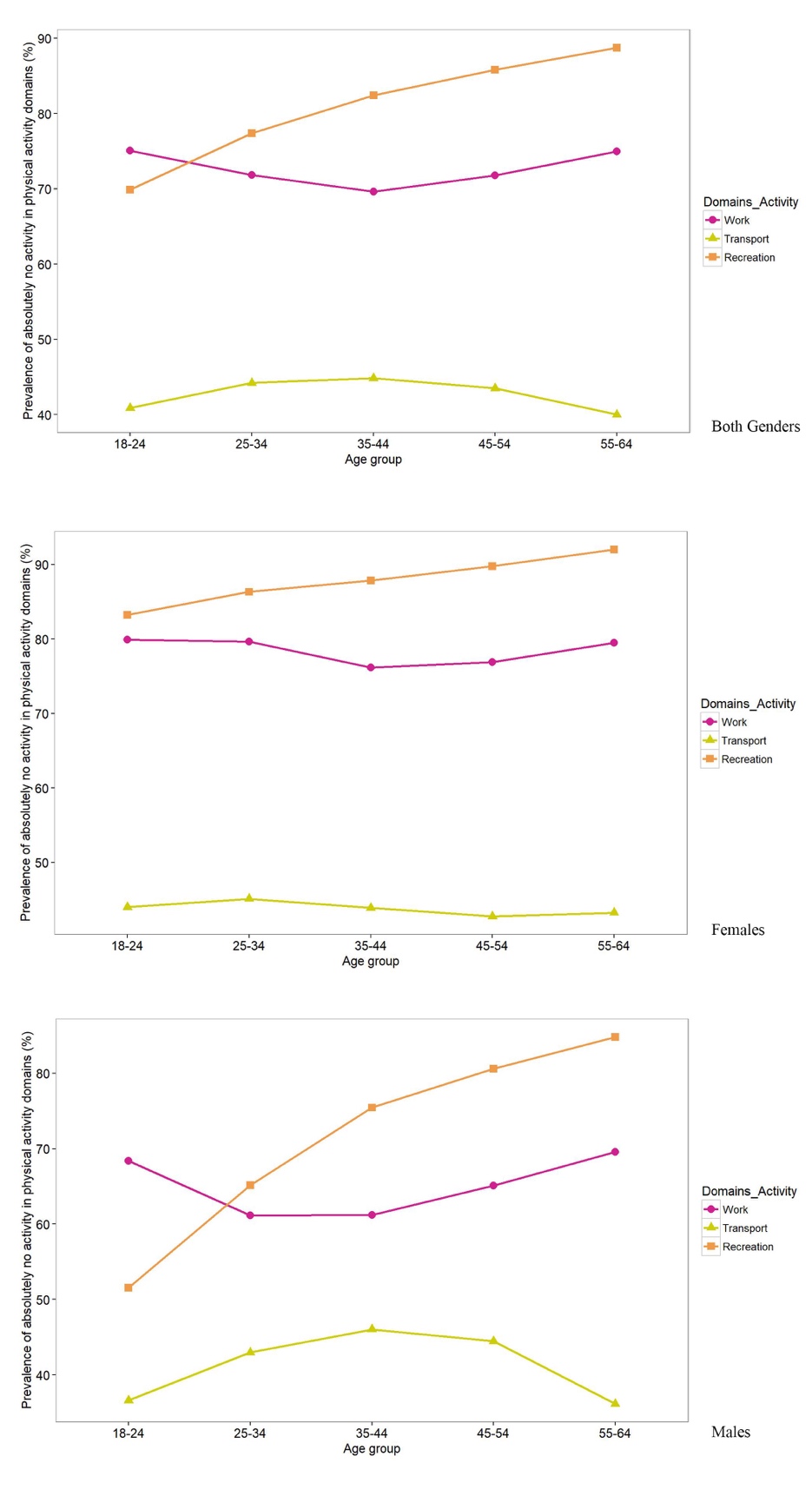


*Figure S1*. Prevalence of absolutely no activity in physical activity domains among adult Iranian gender and age groups.


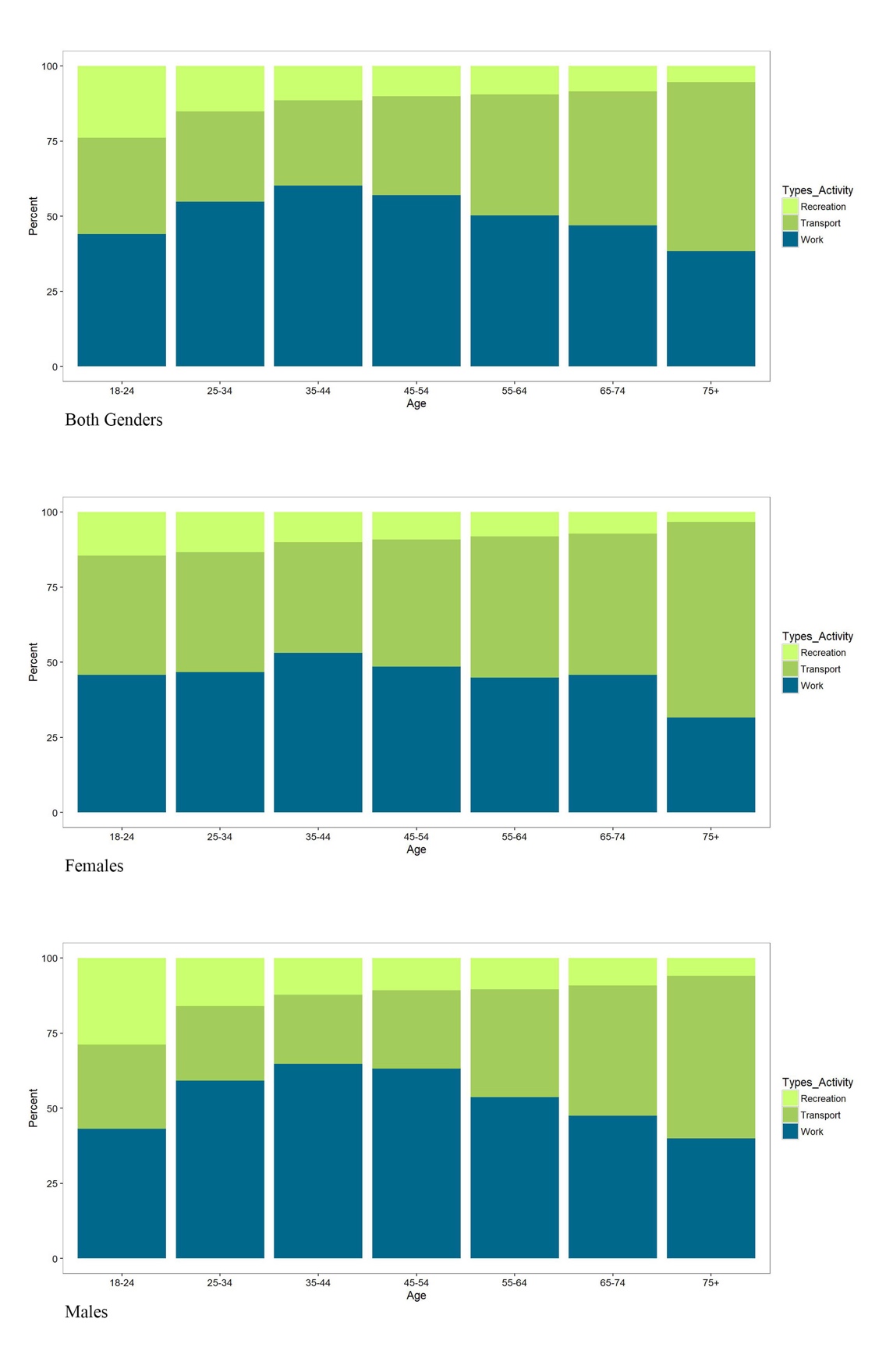


*Figure S2*. Percentage of physical activity domains’ contribution in total METs among adult Iranian gender and age groups.
